# ZSCAN4 interacts with PARP1 to promote DNA repair in mouse embryonic stem cells

**DOI:** 10.1186/s13578-023-01140-1

**Published:** 2023-10-24

**Authors:** Li-Kuang Tsai, Min Peng, Chia-Chun Chang, Luan Wen, Lin Liu, Xiubin Liang, Y. Eugene Chen, Jie Xu, Li-Ying Sung

**Affiliations:** 1 Institute of Biotechnology, National Taiwan University, Taipei, 106 Taiwan, ROC; 2 Center for Advanced Models for Translational Sciences and Therapeutics, University of Michigan Medical School, Ann Arbor, MI 48109 USA; 3 State Key Laboratory of Medicinal Chemical Biology, Department of Cell Biology and Genetics, College of Life Sciences, Nankai University, Tianjin, 300071 China; 4 Center for Developmental Biology and Regenerative Medicine, Taipei, 106 Taiwan, ROC; 5 Center for Biotechnology, National Taiwan University, Taipei, 106 Taiwan, ROC; 6 Agricultural Biotechnology Research Center, Academia Sinica, Taipei, 115 Taiwan, ROC

**Keywords:** ZSCAN4, PARP1, DNA double strand breaks, Mouse embryonic stem cells

## Abstract

**Background:**

In eukaryotic cells, DNA double strand breaks (DSB) are primarily repaired by canonical non-homologous end joining (c-NHEJ), homologous recombination (HR) and alternative NHEJ (alt-NHEJ). Zinc finger and SCAN domain containing 4 (ZSCAN4), sporadically expressed in 1–5% mouse embryonic stem cells (mESCs), is known to regulate genome stability by promoting HR.

**Results:**

Here we show that ZSCAN4 promotes DNA repair by acting with Poly (ADP-ribose) polymerase 1 (PARP1), which is a key member of the alt-NHEJ pathway. In the presence of PARP1, ZSCAN4-expressing mESCs are associated with lower extent of endogenous or chemical induced DSB comparing to ZSCAN4-negative ones. Reduced DSBs associated with ZSCAN4 are abolished by PARP1 inhibition, achieved either through small molecule inhibitor or gene knockout in mESCs. Furthermore, PARP1 binds directly to ZSCAN4, and the second ⍺-helix and the fourth zinc finger motif of ZSCAN4 are critical for this binding.

**Conclusions:**

These data reveal that PARP1 and ZSCAN4 have a protein–protein interaction, and shed light on the molecular mechanisms by which ZSCAN4 reduces DSB in mESCs.

**Supplementary Information:**

The online version contains supplementary material available at 10.1186/s13578-023-01140-1.

## Background

Zinc finger and SCAN domain containing 4 (ZSCAN4) is expressed in two-cell (2C) stage mouse embryos and in the so-called 2C-state mouse embryonic stem cells (mESCs), which is a 1 to 5% subpopulation of the mESCs that exhibit a gene expression pattern similar to that of the totipotent 2C-stage embryos [1, 2]. For this reason, ZSCAN4 is regarded as a Bona-fide marker of 2C-state mESCs.

Maintaining genome stability is essential for early-stage embryos as well as for pluripotent stem cells (PSCs). Double-strand breaks (DSB) are the most lethal form of DNA damage in eukaryotic cells. DSBs are repaired through different pathways, which include canonical non-homologous end join (c-NHEJ), homologous recombination (HR), alternative non-homologous end join (alt-NHEJ), and others [3]. Among them, c-NHEJ and alt-NHEJ are error-prone, and HR leads to precise repair. In 2010, Zalman et al. reported that ZSCAN4 promotes HR in mESCs and elongate telomeres [2]. Later, other groups reported that the level of ZSCAN4 is reversely correlated with the extent of DSB in mouse induced pluripotency stem (iPS) cells [4] and preimplantation embryos [5]. These findings indicate that ZSCAN4 plays a key role in resolving DSBs in embryos and PSCs.

It is less clear how ZSCAN4 gets involved in DSB resolution. Dan et al. suggested that ZSCAN4 may achieve this through modulating the epigenetic status [6]. They demonstrated that ZSCAN4 induces global DNA demethylation through downregulation of ubiquitin like with PHD and ring finger domains 1 (UHRF1) and DNA methyltransferase 1 (DNMT1), major components of the maintenance DNA methylation machinery. Srinivasan et al. demonstrated another possibility that ZSCAN4 binds to DSB-prone sequences (e.g., microsatellite DNA) thereby protects them from breaking under stress [5].

Poly(ADP-ribose) polymerase 1 (PARP1) is a key factor in DNA repair. It is involved in c-NHEJ and HR [7, 8], but most importantly in the alt-NHEJ pathway [9] where it competes with c-NHEJ components such as the KU heterodimer (KU70/KU80) to bind the DSB [10], then recruits DNA ligase 3 (LIG3) and DNA Polymerase theta (POLθ) to complete the alt-NHEJ repair [11–14].

In the present work, we asked the question whether there is any interaction between PARP1 and ZSCAN4 in the DNA repair process. We show that ZSCAN4 reduces DSB in a PARP1-dependent manner. We reveal that there is a protein–protein binding between these two proteins, and we provide motif level resolution of this interaction. Our results suggests that ZSCAN4 engages PARP1 in resolving DSBs in mESCs.

## Results

### ZSCAN4 expression is associated with reduced DSBs in mESCs

**Fig. 1:**
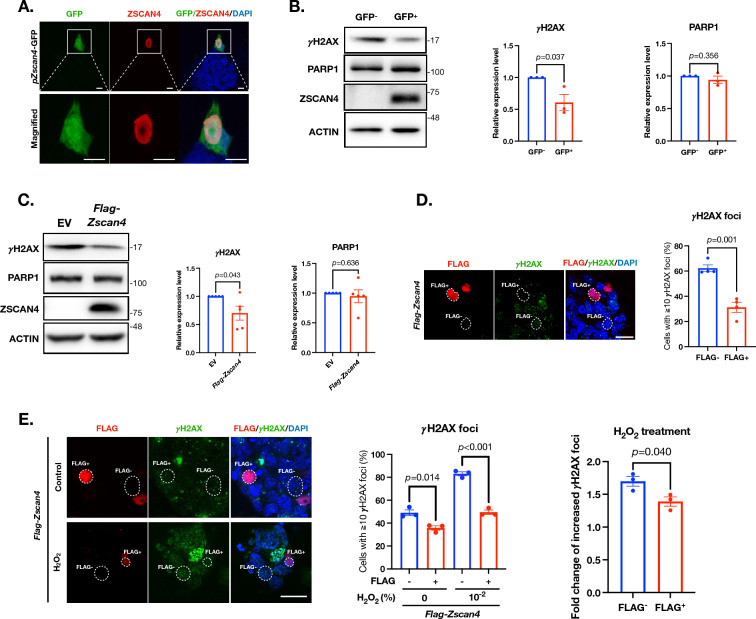
ZSCAN4 expression is reversely correlated with the extent of DSBs in mESCs. **A** Immunofluorescence images of the *pZscan4-GFP* mESCs. Note only a small subpopulation of cells (boxed in top row) express ZSCAN4 at a given time. Scale bar: 10 µm. **B** Left: western blot of γH2AX, PARP1, and ZSCAN4 the GFP^+^ (indicative of ZSCAN4 expressing) and GFP^−^ (indicative of ZSCAN4-negative) mESCs. Middle and right: quantitative levels of γH2AX and PARP1. Data are normalized to the GFP^−^ group and are represented as mean ± SEM. **C** Left: western blot of γH2AX, PARP1, and ZSCAN4 in the wildtype mESCs transiently expressing FLAG-ZSCAN4. Middle and right: quantitative levels of γH2AX and PARP1. Data are normalized to the GFP^−^ group and are represented as mean ± SEM. **D** Left: IF images of FLAG and γH2AX in wildtype mESCs transiently overexpressing FLAG-ZSCAN4. Scale bar: 20 µm. Right: quantitative percentage of cells with ≧10 γH2AX foci. Data are represented as mean ± SEM. **E** Left: IF images of FLAG and γH2AX in wildtype mESCs transiently overexpressing FLAG-ZSCAN4 with 0.01% H_2_O_2_ treatment. Scale bar: 20 µm. Middle: quantitative percentage of cells with ≧10 γH2AX foci. Data are represented as mean ± SEM. Right: fold change of the percentage of cells with ≧10 γH2AX foci after the H_2_O_2_ treatment. See also Additional file 1: Figs. S1, S2 and Table S2

Previous studies show that ZSCAN4 is associated with DSB reduction in mouse iPSCs [4] and preimplantation embryos [5]. To verify these, we established a reporter mESC line that expresses the green fluorescent protein (GFP) driven by the *Zscan4* promoter (*pZscan4-GFP*, Additional file 1: Fig. S1A). Immunofluorescent (IF) images show that there is a faithful co-expression of GFP and ZSCAN4 (Fig. 1A).

We next separated the GFP-expressing (GFP^+^) and GFP-negative (GFP^−^) mESCs by the fluorescence-activated cell sorter (FACS, Additional file 1: Fig. S1B, C) and determined the ZSCAN4 protein levels in these two subpopulations. As expected, a strong ZSCAN4 band is observed in the western blot of the GFP^+^ cell population, but not that of the GFP^−^ cell population (Fig. 1B).

We compared the endogenous extent of DSB between the GFP^+^ and GFP^−^ cells by determining the extent of S139 phosphorylation of H2AX (γH2AX). Upon DSB formation, H2AX, a variant of the H2A protein that is part of the histone octomer in nucleosomes, are quickly phosphorylated to mark the site of damage so that recruitment of DSB repair factors can take place efficiently [15]. The γH2AX thus serve as a faithful indicator of the DSB events in mammalian cells [16–18]. By western blot, we show that γH2AX levels are lower in the GFP^+^ cells than in the GFP^−^ cells (Fig. 1B), reversely correlated with levels of ZSCAN4. Notably, PARP1 are expressed at comparable levels between the GFP^+^ and GFP^−^ subpopulations (Fig. 1B), indicating that levels of ZSCAN4 does not influence the levels of PARP1 in mESCs.

To confirm the observations in the *pZscan4-GFP* stable cell line, we transiently overexpressed ZSCAN4 by lipofectamine mediated transfection of a *Flag-Zscan4* expression plasmid (*pFlag-Zscan4*) to mESCs (Additional file 1: Fig. S2A). Comparing to the cells transfected by an empty vehicle vector (EV), *pFlag-Zscan4* transfected mESCs had a significantly lower level of γH2AX signals in Western blot (Fig. 1C). Levels of PARP1 were comparable between the overexpression and the EV groups (Fig. 1C), consistent with the findings in the *Zscan4*-GFP reporter cells (Fig. 1B). We also employed IF images to quantify the DSB extent by counting and calculating the percentage of cells with ≥ 10 γH2AX foci, as previously reported [19–22]. The FLAG signal was used to identify FLAG-ZSCAN4 positive cells. Approximately 60% cells possessed ≥ 10 γH2AX foci in the FLAG-ZSCAN4 negative cells, which is about twice that in the FLAG-ZSCAN4 positive cells (Fig. 1D), again indicating a role of ZSCAN4 in DSB reduction.

Next, we introduced an exogenous DNA damage reagent, hydrogen peroxide (H_2_O_2_) at 0.01%, to the system to induce DSB formation. In the FLAG-ZSCAN4 negative cells, H_2_O_2_ treatment increased the percentage of cells with ≥ 10 γH2AX foci by 1.7-fold, from 49.08 ± 2.49% to 83.04 ± 1.76% (Fig. 1E, left and middle panels). In the FLAG-ZSCAN4 positive cells, H_2_O_2_ treatment also increased the percentage of cells with ≥ 10 γH2AX foci, but to a lesser extent, by 1.4-fold from 35.77 ± 1.95% to 49.52 ± 1.90% (Fig. 1E). This result suggests a protective role of ZSCAN4 against exogenous DSB generating agents.

Together, these data show that ZSCAN4 is reversely associated with DSB signal levels in mESCs.

### ZSCAN4-associated DSB resolution capacity engages PARP1

It has been reported that ZSCAN4 promotes HR [2]. It is not known whether other DNA repair pathways, such as alt-NHEJ, plays a role in ZSCAN4-associated DSB reduction.

To investigate this, we treated *pFlag-Zscan4* transduced mESCs with a small molecule compound 3-Aminobenzamide (3-AB). 3-AB is a potent inhibitor of PARP and is commonly used to suppress the alt-NHEJ pathway [23].

**Fig. 2:**
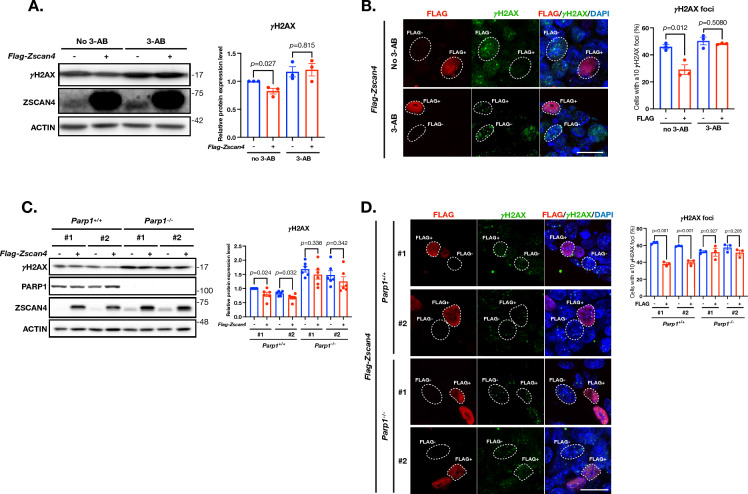
ZSCAN4-associated DSB reduction is dependent on PARP1. **A** Left: western blot of γH2AX and ZSCAN4 in wildtype mESCs transiently overexpressing FLAG-ZSCAN4 treated with or without 3-AB. 0.1% DMSO serves as the vehicle control (no 3-AB). Right: quantitative levels of γH2AX. Data are normalized to no 3-AB (control) group and are represented as mean ± SEM. **B** IF images of FLAG and γH2AX in wildtype mESCs transiently overexpressing FLAG-ZSCAN4 treated with or without 3-AB. Scale bar: 20 µm. Right: quantitative percentage of cells with ≧10 γH2AX foci. Data are represented as mean ± SEM. **C** Left: western blot of γH2AX, PARP1 and ZSCAN4 in wildtype (*Parp1*^+*/*+^) and PARP1 knockout (*Parp1*^*−/−*^) mESCs. For each genotype, two clones (#1 and #2) were used. Right: quantitative levels of γH2AX. Data are normalized to clone #1 of *Parp1*^+*/*+^ mESCs and are represented as mean ± SEM. (D) Left: IF images of FLAG and γH2AX in wildtype (*Parp1*^+*/*+^) and PARP1 knockout (*Parp1*^*−/−*^) mESCs. Right: quantitative percentage of cells with ≧10 γH2AX foci. Data are represented as mean ± SEM. See also Figure S2, S3 and Additional file 1: Table S2

Western blot analysis revealed that without 3-AB, the γH2AX signals were, as expected and consistent with earlier results, significantly lower in the *pFlag-Zscan4* transfected cells than those transfected with the EV (Fig. 2A). Intriguingly, with 3-AB, the γH2AX signal levels became similar between these two groups, while both are higher than those without 3-AB (Fig. 2A). The γH2AX foci counting assay by IF confirmed this observation (Fig. 2B). These findings indicated that PARP inhibition not only increased the overall DSB extent, but also eliminated the DSB reduction effect associated with ZSCAN4, suggesting that PARP proteins may participate in the ZSCAN4-associated DSB resolution.

3-AB is a general inhibitor of PARP including PARP1 and PARP2. To delineate if PARP1 participates in ZSCAN4-associated DSB resolution, we generated *Parp1* knockout (KO) mESCs by CRISPR/Cas9 (Additional file 1: Fig. S3). Two *Parp1* KO (*Parp1*^*−/−*^) mESC clones (#1 and #2) were selected and maintained for the following experiments. Both KO lines had no detectable PARP1 protein expression as evidenced by Western blot (Fig. 2C). The DSB extent, indicated by the γH2AX bands in the Western blot, was much higher in the KO lines than in the *Parp1* wildtype cells (Fig. 2C), which is as expected because *Parp1* is a key factor in several DNA repair pathways especially in the alt-NHEJ pathway. We next checked if *Parp1* KO had any effects on ZSCAN4’s DSB reduction capacity. Both Western blot and γH2AX foci counting assays indicated so: in the *Parp1* wildtype mESCs, the extent of DSB is significantly lower in the *pFlag-Zscan4* transfected cells than in EV transfected cells or FLAG-ZSCAN4 negative cells; in the *Parp1*^*−/−*^ mESCs, the extent of DSB became similar between these two subpopulations (Fig. 2C and D). This observation suggests that PARP1 contributes to ZSCAN4-associated DSB resolution.

Taken together, our results indicate that ZSCAN4 engages PARP1 in resolving DSB in mESCs.

### PARP1 has a protein–protein interaction with ZSCAN4

**Fig. 3:**
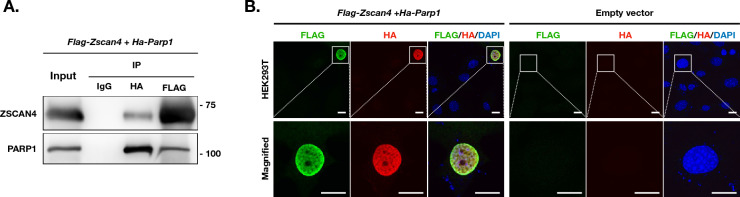
PARP1 has a protein–protein interaction with ZSCAN4. **A** Co-IP analysis of FLAG-ZSCAN4 and HA-PARP1 expression plasmids in HEK293T cells. **B** IF images of FLAG-ZSCAN4 and HA-PARP1 expression plasmids in HEK293T cells. Scale bar: 15 µm. See also Additional file 1: Fig. S4

Given the potential role of PARP1 in ZSCAN4-associated DSB resolution, we asked the question whether ZSCAN4 and PARP1 proteins directly interact. We constructed *Flag-Zscan4* and *Ha-Parp1* overexpression plasmids (Additional file 1: Fig. S2A and B) and co-transfected them into HEK293T cells. We examined the interaction of ZSCAN4 with PARP1 by co-immunoprecipitation (co-IP). Protein complexes were isolated using an anti-HA antibody, then blotted with ZSCAN4 or FLAG antibodies (Fig. 3A and Additional file 1: Fig. S4). The results show that PARP1 pulled down ZSCAN4 (Fig. 3A and Additional file 1: Fig. S4). IgG was used as a control which yielded no positive signal in immunoprecipitations (IPs). Using a reverse IP/immunoblotting (IB) protocol, IPs were performed by anti-FLAG antibody, then blotted with PARP1 or HA antibodies. We confirmed that ZSCAN4 pulled down PARP1 (Fig. 3A and Additional file 1: Fig. S4). In line with this finding, IF images revealed that FLAG-ZSCAN4 and HA-PARP1 co-localized in the nucleus (Fig. 3B). These observations suggest that there is a protein–protein interaction between ZSCAN4 and PARP1.

**Fig. 4:**
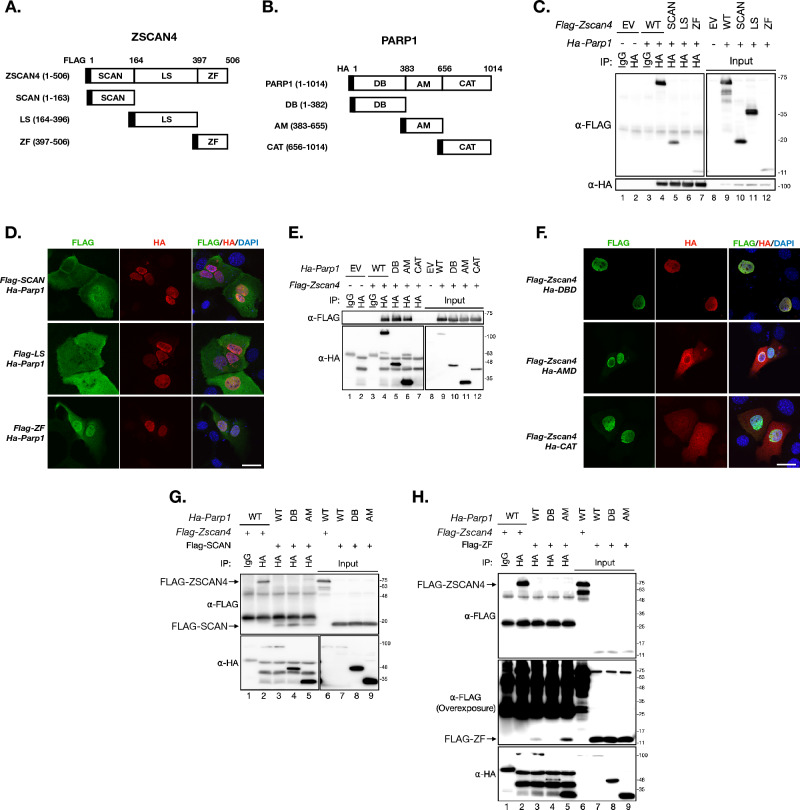
ZSCAN4 and PARP1 interacts with each other. **A** The illustration of ZSCAN4 protein domains. The numbers on the top indicate the residue positions. SCAN: the SCAN domain (amino acid residue 1–163 of ZSCAN4); LS: the linker sequence (amino acid residue 164–396 of ZSCAN4); ZF: the zinc finger domain (amino acid residue 397–506 of ZSCAN4). **B** The illustration of PARP1 protein domains. DB: the DNA binding domain (amino acid residue 1–382 of PARP1); AM: the auto-modification domain (amino acid residue 383–655 of PARP1); CAT: the catalytic domain (amino acid residue 656–1014 of PARP1). **C** Co-IP of individual FLAG-ZSCAN4 domains (SCAN, LS or ZF) and full-length HA-PARP1. **D** IF images of FLAG (indicative of ZSCAN4 domains) and HA (indicative of full length PARP1) in mouse BNL CL.2 transiently expressing full length HA-PARP1 and a FLAG tagged ZSCAN4 domain. Scale bar: 25 µm. **E** Co-IP of individual HA-PARP1 domains (DB, AM or CAT) and full-length FLAG-ZSCAN4. **F** IF images of FLAG (indicative of full length ZSCAN4) and HA (indicative of PARP1 domains) in mouse BNL CL.2 transiently expressing full length FLAG-ZSCAN4 and a HA tagged PARP1 domain. Scale bar: 25 µm. **G** Co-IP of the HA-DB and HA-AM (of PARP1) with FLAG-SCAN (of ZSCAN4). The top arrow indicates the full-length FLAG-ZSCAN4 bands. The lower arrow indicates the FLAG-SCAN domain (of ZSCAN4) bands. WT: wildtype. **H** Co-IP results of the HA-DB and HA-AM (of PARP1) with FLAG-ZF (of ZSCAN4). The top arrow indicates the full-length FLAG-ZSCAN4 bands. The lower arrow indicates the FLAG-ZF domain (of ZSCAN4) bands. The middle panel is an overexposure of the top panel to reveal the FLAG-ZF bands. WT: wildtype

To dissect this protein–protein interaction, we constructed plasmids to express different versions of truncated ZSCAN4 and PARP1 (Fig. 4A and B). For truncated ZSCAN4, we designed three plasmids, each expressing one of the following: the SCAN domain only (SCAN, 1–163 residues), the linker sequence only (LS, 164–396 residues), and the Zinc finger domain only (ZF, 397–506 residues). In each plasmid, a *Flag* tag sequence was added to the N-terminal for antibody detection (Fig. 4A). For truncated PARP1, we also designed three plasmids, each expressing one of the following: the DNA binding domain only (DB, 1-382 residues), the auto-modification domain only (AM, 383-655 residues), and the catalytic domain only (CAT, 656-1014 residues). In each plasmid, an *Ha* tag sequence was added for antibody detection (Fig. 4B).

To identify the key PARP1-binding domain on ZSCAN4, we co-transfected the cells with plasmids that express (i) the full length PARP1 and (ii) one of the truncated ZSCAN4. IP results suggest that ZSCAN4 interacts with PARP1 through its SCAN and ZF domains, but not the LS domain (Fig. 4C, lanes 5 and 7). IF images confirmed the findings from the IP experiments (Fig. 4D).

Likewise, to identify the key ZSCAN4-binding domain on PARP1, we co-transfected the cells with plasmids that express (i) the full length ZSCAN4 and (ii) one of the truncated PARP1. IP results show that the DB and AM domains, but not the CAT domain of PARP1, participated in the binding with ZSCAN4 (Fig. 4E, lanes 5 and 6). IF images confirmed the findings from the IP experiments (Fig. 4F).

After knowing that the SCAN and ZF domains from ZSCAN4 (designated as z-SCAN and z-ZF to indicate its protein origin) and the DB and AM domains from PARP1 (designated as p-DB and p-AM) participate in the protein–protein binding between these two proteins, we next looked at the binding relationships between these individual domains. All four combinations of these domain expression plasmids, (i) z-SCAN + p-DB; (ii) z-SCAN + p-AM; (iii) z-ZF + p-DB; and (iv) z-ZF + p-AM, were transfected into HEK293T cells, followed by IP to exam if there are any direct binding between the two corresponding domains (Additional file 1: Table S1). The results indicated that the z-SCAN domain bind to either the p-DB or the p-AM domain (Fig. 4G, lanes 4 and 5); whereas the z-ZF domain only binds with the p-AM but not the p-DB domain (Fig. 4H, lane 5).

**Fig. 5:**
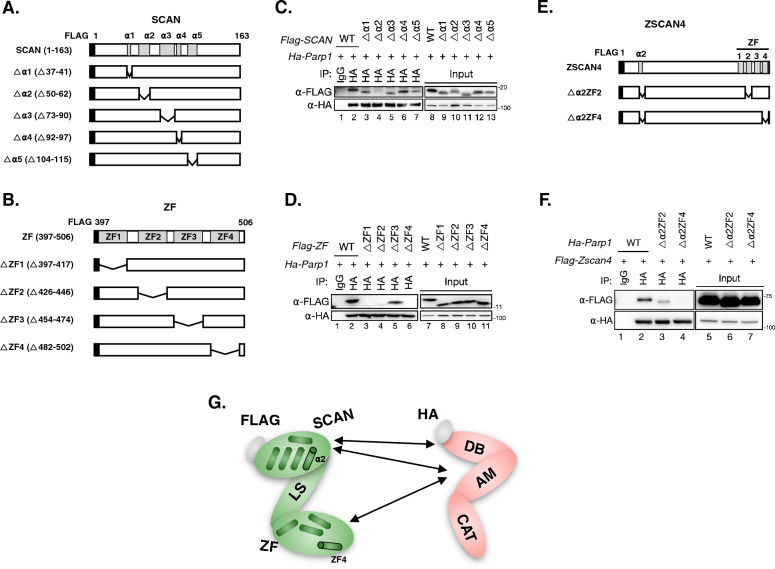
The ⍺2 motif on the SCAN domain and the ZF4 motif on the ZF domain are critical for the binding of ZSCAN4 with PARP1. **A** Illustration of the SCAN domain (top) and its truncated variants (△⍺1 to △⍺5). **B** Illustration of the ZF domain (top) and its truncated variants (△ZF1 to △ZF4). **C** Co-IP of the full and truncated FLAG-SCAN domain with the full-length HA-PARP1. **D** Co-IP of the full and truncated FLAG-ZF domain with the full-length HA-PARP1. **E** Illustration of the full-length FLAG-ZSCAN4 and two truncated variants (△⍺2ZF2 and △⍺2ZF4). **F** Co-IP of FLAG-ZSCAN4 (full length), △⍺2ZF2 and △⍺2ZF4 (truncated variants) with the full-length HA-PARP1. **G** Illustration of the identified interactions between different motifs of ZSCAN4 and PARP1. The arrows indicate inter-domain bindings. The ⍺2 motif on the SCAN domain and the ZF4 motif are labeled to highlight their importance. See also Additional file 1: Table S1

Prior studies have revealed that there are 5 ⍺-helixes (⍺1 to ⍺5) on the z-SCAN domain and 4 zinc finger motifs (ZF1 to ZF4) on the z-ZF domain [6, 24]. To gain insight on the interaction at the motif resolution of ZSCAN4, we next constructed truncated z-SCAN domain expression plasmids each missing one of the ⍺-helixes (△⍺1 to △⍺5, Fig. 5A), and truncated z-ZF domain expression plasmids each missing one of the ZF motifs (△ZF1 to △ZF4, Fig. 5B). These truncated domain plasmids (tagged with *Flag*) were individually co-transfected with full length HA-PARP1 expression plasmid for IP experiments (Additional file 1: Table S1). The results show that the deletion of the ⍺2 of the z-SCAN domain (Fig. 5C, lane 4), or the deletion of the ZF1, ZF2 or ZF4 of the z-ZF domain (Fig. 5D, lanes 3, 4, and 6) abolished the interaction of the corresponding domain with the full-length HA-PARP1. As such, the ⍺2 motif on the z-SCAN domain, and the ZF1, ZF2 and ZF4 motifs on the z-ZF domain are potentially essential for ZSCAN4 to establish the protein–protein interaction with PARP1.

To verify these results, we constructed two mutant ZSCAN4 expression plasmids: (i) missing the ⍺2 and the ZF2 motifs (△⍺2ZF2); and (ii) missing the ⍺2 and the ZF4 motifs (△⍺2ZF4). We co-transfected the △⍺2ZF2 or the △⍺2ZF4 plasmid with full length HA-PARP1 expression plasmid for IP experiments (Fig. 5E). As expected, both mutations (△⍺2ZF2 and △⍺2ZF4) compromised the binding between ZSCAN4 and PARP1. Strikingly, the △⍺2ZF4 mutation totally abolished the IP signals between ZSCAN4 and PARP1 (Fig. 5F, lane 4). We summarize the interacting domains of ZSCAN4 and PARP1 in the Fig. 5G.

**Fig. 6:**
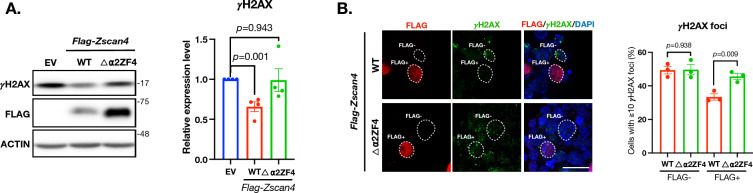
The ⍺2 and ZF4 motifs of ZSCAN4 are required for ZSCAN4-associated DSB reduction. **A** Left: western blot of γH2AX and FLAG (indicative of full length or truncated ZSCAN4) in wildtype mESCs transfected with plasmids expressing either the full length (WT) or truncated (△⍺2ZF4) ZSCAN4. EV: empty vector. Right: quantitative levels of γH2AX. Data are normalized to EV transfected mESCs and are represented as mean ± SEM. **B** Left: IF images of FLAG and γH2AX in wildtype mESCs transfected with plasmids expressing either the full length (WT) or truncated (△⍺2ZF4) ZSCAN4. Right: quantitative percentage of cells with ≧10 γH2AX foci. Data are represented as mean ± SEM. See also Additional file 1: Table S2

We then transfected the mESCs with the △⍺2ZF4 ZSCAN4 plasmid to investigate the effect of this mutant on the ZSCAN4-associated DSB resolution. Both the Western blot and γH2AX foci assays show that this mutation, presumptively through the disruption of the ZSCAN4-PARP1 protein–protein-interaction, abolished the DSB-reduction capacity associated with WT ZSCAN4 (Fig. 6A, B).

Together, these results reveal that PARP1 interacts with ZSCAN4. The ⍺2 motif on the SCAN domain and the ZF4 motif on the ZF domain are essential for the binding. The binding between these two proteins is essential for ZSCAN4-associated DSB resolution capacity.

## Discussion

It has been over a decade since the first report on ZSCAN4’s role in promoting HR. It remains to be fully elucidated, however, how ZSCAN4 is involved in the DNA repair process. It was shown that ZSCAN4 reduces DNA methylation, which is HR promoting, through UHRF1 mediated degradation of DNA methyltransferase DNMT1 [6]. In another work, it suggests that ZSCAN4 binds to DSB-prone sequences (e.g., microsatellite DNA) thereby protects them from breaking under stress [5].

We present a new mechanism that ZSCAN4’s DSB resolution capacity is PARP1 dependent. Inhibiting PARP1 abolishes the DSB-reduction benefits associated with ZSCAN4. Therefore, besides HR, ZSCAN4 is potentially also involved in the alt-NHEJ pathway. Furthermore, ZSCAN4 and PARP1 has a protein–protein binding interaction. It is known that PARP1 is engaged in the alt-NHEJ process at multiple points. For example, the early resection factor MRE11 is recruited by PARP1 [25]. PARP1 also plays the key role in recruiting LIG3, the ultimate molecule for the last ligation step in alt-NHEJ [26, 27]. It is possible that ZSCAN4 interacts with PARP1 in one or more of these steps. Future studies are warranted to dissect this.

How the interaction between ZSCAN4 and PARP1 contributes to the DSB resolution is intriguing. Based on the current data, we speculate at least two possibilities. First, ZSCAN4’s binding with PARP1 could bring alt-NHEJ repair factors to the DSB lesion. This may be particularly probable at DSB-vulnerable loci where ZSCAN4 reportedly bind to [5]. The binding between ZSCAN4 and PARP1 there (if any) would allow a quick assembly of alt-NHEJ repair factors to resolve any emerging DSBs. Another possibility is that PARP1 is a regulator of ZSCAN4. This could be achieved at the protein level through the interaction between PARP1 mediated PARylation and ZSCAN4. ZF4 motif of ZSCAN4, which was found to interact with PARP1 in this study, contains a consensus PAR interacting motif [28]. This regulation may also be achieved at the transcription level. There is a PARP1 binding sequence in the human *ZSCAN4* promoter [29]. The same binding consensus is found in mouse *Zscan4* promoter (GGAAAGG), suggesting that PARP1 may directly bind on *Zscan4* promoter to regulate its expression level.

Our work also provides insight into telomere regulation in mESCs by ZSCAN4. ZSCAN4 is known for maintaining telomere length by promoting the HR-based alternative lengthening of telomeres (ALT) pathway [2, 6]. PARP1, on the other hand, repairs telomere DSBs through the alt-NHEJ pathway. These two seemingly independent telomere DSB resolution pathways may now be linked given the potential interaction between ZSCAN4 and PARP1. PARP1 should be considered in efforts to modulate telomeres by ZSCAN4, and vice versa. It should be further noted that ZSCAN4 and PARP1 are both reported to promote iPSC reprogramming [4, 30]. Our work suggests that ZSCAN4 and PARP1 may have worked as a pair to improve iPSC reprogramming by preventing or repairing DNA damage induced in the rapid iPSC reprogramming process. Modulating the interaction between ZSCAN4 and PARP1 may represent a new route to improve the quality of iPSCs, as well as other stem cell types, for example, ESCs, that express ZSCAN4.

## Conclusions

We reveal a protein-to-protein interaction between ZSCAN4 and PARP1 at the motif resolution. We demonstrate that PARP1 participates in ZSCAN4 mediated DSB repair in mESCs. These data provide novel insights on ZSCAN4 and PARP1 biology.

## Materials and methods

### Animals

The animals used in this project were maintained, cared, and used according to the animal protocol #NTU-105-EL-164 that was reviewed and approved by the Institutional Animal Care and Use Committee (IACUC) of National Taiwan University.

### Mouse embryonic stem cells

The wildtype mESCs were derived from blastocyst embryos collected from fertilized superovulated female mice following our routine protocol [31].

To generate the p*Zscan4*-GFP mESCs, the *Zscan4* promoter sequence cloned from 2570 bp upstream of *Zscan4c* start codon [2] and a 720 bp eGFP coding sequence were cloned into the pSin vector (16578, Addgene) and the plasmid was transfected into HEK293T cell along with pSPAX2 (12260, Addgene) and pMD2.G (12259, Addgene) to produce lentivirus. Conditioned medium containing lentivirus was harvested and used to treat the wildtype mESCs, followed by FACS to enrich the pZscan4-GFP cells for subsequent culture.

To generate the *Parp1* knockout mESC lines, we designed a guide RNA (gRNA, 5′-CTGGTACCATCCAACTTGCT-3′) targeting Exon 4 of the *Parp1* gene. The gRNA was cloned to the Cas9 expressing plasmid (64221, Addgene) containing a mCherry reporter, following a reported CRISPR/Cas9 protocol [32]. We constructed a homologous recombination (HR) template containing a T2A-eGFP-stop codon sequence flanked by 1003 bp long homology arms on each side (Additional file 1: Fig. S3B). The Cas9 and HR template plasmids were transfected to mESCs by the lipofectamine stem transfection reagent (STEM00015, Thermo). 24 h after transfection, GFP and mCherry double positive mESCs were sorted out by FACS and single cell seeded in the 96-well plate to derive the KO clones. PCR (forward primer: GCCAGATGCGCCTGTCCA; reverse primer: TTCTTGATGGCCGGGAGCT) was performed to confirm the successful insertion.

The wildtype, p*Zscan4*-GFP and the Parp1 KO mESCs were all cultured in Dulbecco's modified Eagle's medium (DMEM; 11965084, Thermo, Carlsbad, CA, USA) with 15% fetal bovine serum (FBS; TMR-016-B, Millipore, Darmstadt, Germany) supplemented with 1% Penicillin/Streptomycin Solution (P/S; 15140122, Thermo), 2 mM GlutaMax (35050061, Thermo), 0.1 mM nonessential amino acids (11140-050, Thermo), 0.1 mM 2-mercaptoethanol (ES-007-E, Millipore), 1 mM sodium pyruvate (11,360,070, Thermo) and 1000 units/mL Leukemia Inhibitory Factor (ESG1107, Millipore). Mitomycin C (2 μg/mL M4287, MilliporeSigma, Burlington, MA, USA) treated E13.5 mouse embryonic fibroblast (MEF) cells were used as the feeder cells for mESC culture.

### HEK293T and BNL CL.2 cells

Human HEK293T (CRL-3216, ATCC, Manassas, VA, USA) and mouse BNL CL.2 (TIB-73, ATCC) cells were cultured in DMEM (11965084, Thermo) with 10% FBS (TMR-016-B, Millipore) supplemented with 1% P/S (15140122, Thermo). Plasmid transfection to these cells was performed by JetPrime (101000046, Polyplus, Illkirch-Graffenstaden, Bas-Rhin, France) following the manufacture’s instruction.

### 3-Aminobenzamide

3-Aminobenzamide (3-AB, A0788, MilliporeSigma) was dissolved in dimethyl sulfoxide (DMSO, D2650, MilliporeSigma) to the final concentration of 10 M as the stock solution. The stock solution was added to the culture medium at 2000 dilution to reach a working concentration of 5 mM 3-AB.

### Immunofluorescence (IF) staining

Cells on cover slides were fixed with 10% formaldehyde (MA-H121-08, Crespellano, Italy). 2% bovine serum albumin (BSA, A9647, MilliporeSigma) and 0.25% Triton-X-100 (X100, MilliporeSigma) in phosphate-buffered saline (PBS, IB3012, Omics Bio, Taipei, Taiwan) was used for permeabilizing cells before they were incubated with the primary antibodies overnight at 4 ℃ followed by secondary antibodies and DAPI for 2 h at room temperature. The antibodies used were ZSCAN4 (ab4340, Millipore), FLAG (F7425, MilliporeSigma), FLAG (66008-4-Ig, Proteintech, Rosemont, IL, USA), γH2AX (ab2893, Abcam, Cambridge, UK), HA (sc-7392, Santa Cruz, Dalla, TX, USA), Alexa anti-mouse 488 (A11001, Thermo), Alexa anti-rabbit 488 (A11034, Thermo), Alexa anti-mouse 594 (A11032, Thermo), and Alexa anti-rabbit 647 (A27040, Thermo). The images were captured by the laser-scanning confocal microscope (TCS SP5 II confocal microscope, Leica, Wetzlar, Germany).

### γH2AX foci counting

To count the number of γH2AX foci, images obtained from confocal microscopy were analyzed by the ImageJ software [33] (exampled in Additional file 1: Fig. S5). The counted number of cells in each experiment (range from 129 to 846) were listed in Additional file 1: Table S2.

### Immunoprecipitation

Immunoprecipitation (IP) was performed by using the Dynabeads protein G IP kit (10007D, Thermo), following the manufacturer’s instruction. Briefly, HEK293T cells transfected with epitope-tagged expression plasmid(s) were lysed in the RIPA buffer (92590, Millipore) for 10 min at 4 ℃ and centrifuged at 16,000×*g* for supernatant collection. The Dynabeads were incubated with 4 μg HA antibody (sc-7392, Santa Cruz) or 2 μg FLAG antibody (F7425, MilliporeSigma) at room temperature for 10 min. Next, the Dynabeads were incubated with 500 µg cell lysate at 4 ℃ for 2 h. After washing, the IP samples were collected and used for Western blot (see next session) to complete the Co-IP assay. Appropriate host of IgG served as control which include mouse IgG (550878, BD, Franklin Lakes, NJ, USA) and rabbit IgG (550875, BD).

### Western blot

For western with IP samples (see previous session), we included the input control which consists of 1% cell lysate. For regular western, 30 µg protein lysate from each sample was used.

Samples were run in electrophoresis using 10% acrylamide gels and then transfer to 0.22 µm PVDF membrane (GE10600021, Millipore). 5% skim milk in TBST (0.1% Tween 20 in TBS) was used to block the membrane for 30 min at room temperature. The membrane was immunoblotted with the primary antibody overnight at 4 ℃ then incubated with HRP-conjugated secondary anti-mouse antibody (31430, Thermo) or HRP-conjugated secondary anti-rabbit antibody (31460, Thermo) for 2 h at room temperature. The signal was detected by T-Pro LumiFast Plus Chemiluminescent Substrate Kit (JT96-K002, T-pro, New Taipei, Taiwan) and captured by GeneGnome XRQ Chemiluminescence with CCD (SynGene, Cambridge, UK). The primary antibodies included FLAG antibody (F7425, MilliporeSigma), HA (sc-7392, Santa Cruz), γH2AX (ab2893, Abcam), ZSCAN4 (ab4340, Millipore), and PARP1 (9542, Cell Signaling, Danvers, MA, USA).

### Vector construction

Plasmids were constructed by the Gibson Assembly Master Mix (E2611L, New England BioLabs, Ipswich, MA, USA) using the pSin vector (16578, addgene, Watertown, MA, USA).

### Statistical analysis

All quantitative data were represented as mean ± standard error of the mean (SEM), with at least 3 biological independent replicates. The statistical comparison between two groups was conducted by unpaired two-tailed student’s t-test (Numbers, Apple, Cupertino, CA, USA).

### Supplementary Information

**Additional file 1: Figure S1.** Generation of the pZscan4-GFP mESCs. (A) The plasmid map of the *pZscan4-*GFP construct. (B) Illustration of the strategy for p*Zscan4*-GFP mESCs generation. (C) Representative FACS plots to isolate GFP + mESCs after pZscan4-GFP plasmid transfection. **Figure S2.** Plasmid maps of FLAG-ZSCAN4 and HA-PARP1. **Figure S3.** *Parp1* knockout in mESCs. (A) The map of Parp1 targeting *Cas9* plasmid. The gRNA (CTGGTACCATCCAACTTGCT) was under U6 promoter. *Cas9* was controlled by chicken β-actin promoter followed by *T2A* and *mCherry*. (B) The map of homologous template for *Parp1* knockout (KO) plasmid. A T2A-GFP-Stop sequence was designed for insertion. (C) Illustration of the *Parp1* knockout strategy. (D) PCR confirmation of the PARP1 knockout. Wildtype (WT) cells had the 208 bp band, whereas cells with the successful knock-in of *T2A-GFP-stop* sequence (which leads to Parp1 KO) had the 993 bp band. WT mESCs and HR template plasmid (PLA) served as control. NC: negative control, water only. **Figure S4.** PARP1 binds with ZSCAN4. Co-IP results of FLAG-ZSCAN4 and HA-PARP1. **Figure S5.** Illustration of the γH2AX foci counting assay. The γH2AX foci (green dots) were counted for each cell. In Example 1, the cell has 7 foci, which is smaller than 10. In Example 2, the cell has 7 foci, which is also smaller than 10. In Example 3, the cell has 23 foci, which is greater than 10. Orange squares indicate the foci counted for the data. Scale bar: 10 µm. **Table S1.** Summary of Co-IP results. **Table S2.** Summary of counted cell numbers in the experiments. **Table S3.** List of antibodies.

## Data Availability

All data analyzed in this study are provided in this article and its additional files. All data used in this study are available from the corresponding author on reasonable request.

## Abbreviations

DSB: DNA double strand breaks
C-NHEJ: Canonical non-homologous end joining
HR: Homologous recombination
Alt-NHEJ: Alternative non-homologous end joining
ZSCAN4: Zinc finger and SCAN domain containing 4
MESCs: Mouse embryonic stem cells
PARP1: Poly (ADP-ribose) polymerase 1
PSCs: Pluripotent stem cells
iPS: Induced pluripotency stem
LIG3: DNA ligase 3
GFP: Green fluorescent protein
IF: Immunofluorescent
FACS: Fluorescence-activated cell sorter
γH2AX: S139 phosphorylation of H2AX
H_2_O_2_: Hydrogen peroxide
3-AB: 3-Aminobenzamide
KO: Knockout
Co-IP: Co-immunoprecipitation
IB: Immunoprecipitations/immunoblotting
SCAN: SCAN domain
LS: Linker sequence
ZF: Zinc finger domain
DB: DNA binding domain
AM: Auto-modification domain
CAT: Catalytic domain
