# Supplementary material for: ZSCAN4 interacts with PARP1 to promote DNA repair in mouse embryonic stem cells
Source: Cell Biosci. 2023 Oct 24;13:193. doi: 10.1186/s13578-023-01140-1 (PMC10594928; doi:10.1186/s13578-023-01140-1)
Supplement: Supplementary file 1 — Additional file 1: Figure S1. Generation of the pZscan4-GFP mESCs. (A) The plasmid map of the pZscan4-GFP construct. (B) Illustration of the strategy for pZscan4-GFP mESCs generation. (C) Representative FACS plots to isolate GFP + mESCs after pZscan4-GFP plasmid transfection. Figure S2. Plasmid maps of FLAG-ZSCAN4 and HA-PARP1. Figure S3. Parp1 knockout in mESCs. (A) The map of Parp1 targeting Cas9 plasmid. The gRNA (CTGGTACCATCCAACTTGCT) was under U6 promoter. Cas9 was controlled by chicken β-actin promoter followed by T2A and mCherry. (B) The map of homologous template for Parp1 knockout (KO) plasmid. A T2A-GFP-Stop sequence was designed for insertion. (C) Illustration of the Parp1 knockout strategy. (D) PCR confirmation of the PARP1 knockout. Wildtype (WT) cells had the 208 bp band, whereas cells with the successful knock-in of T2A-GFP-stop sequence (which leads to Parp1 KO) had the 993 bp band. WT mESCs and HR template plasmid (PLA) served as control. NC: negative control, water only. Figure S4. PARP1 binds with ZSCAN4. Co-IP results of FLAG-ZSCAN4 and HA-PARP1. Figure S5. Illustration of the γH2AX foci counting assay. The γH2AX foci (green dots) were counted for each cell. In Example 1, the cell has 7 foci, which is smaller than 10. In Example 2, the cell has 7 foci, which is also smaller than 10. In Example 3, the cell has 23 foci, which is greater than 10. Orange squares indicate the foci counted for the data. Scale bar: 10 µm. Table S1. Summary of Co-IP results. Table S2. Summary of counted cell numbers in the experiments. Table S3. List of antibodies. [file 13578_2023_1140_MOESM1_ESM.docx]

**Supplemental Information**

**Title**:

ZSCAN4 interacts with PARP1 to promote DNA repair in mouse embryonic stem cells

**Authors**:

Li-Kuang Tsai^1^, Min Peng^1^, Chia-Chun Chang^1^, Luan Wen^2^, Lin Liu^3^, Xiubin Liang^2^, Y. Eugene Chen^2^, Jie Xu^2,*^, Li-Ying Sung^1, 4, 5, 6, *^

**Figure S1. Generation of the pZscan4-GFP mESCs.** (A) The plasmid map of the *pZscan4-*GFP construct. (B) Illustration of the strategy for p*Zscan4*-GFP mESCs generation. (C) Representative FACS plots to isolate GFP+ mESCs after pZscan4-GFP plasmid transfection.


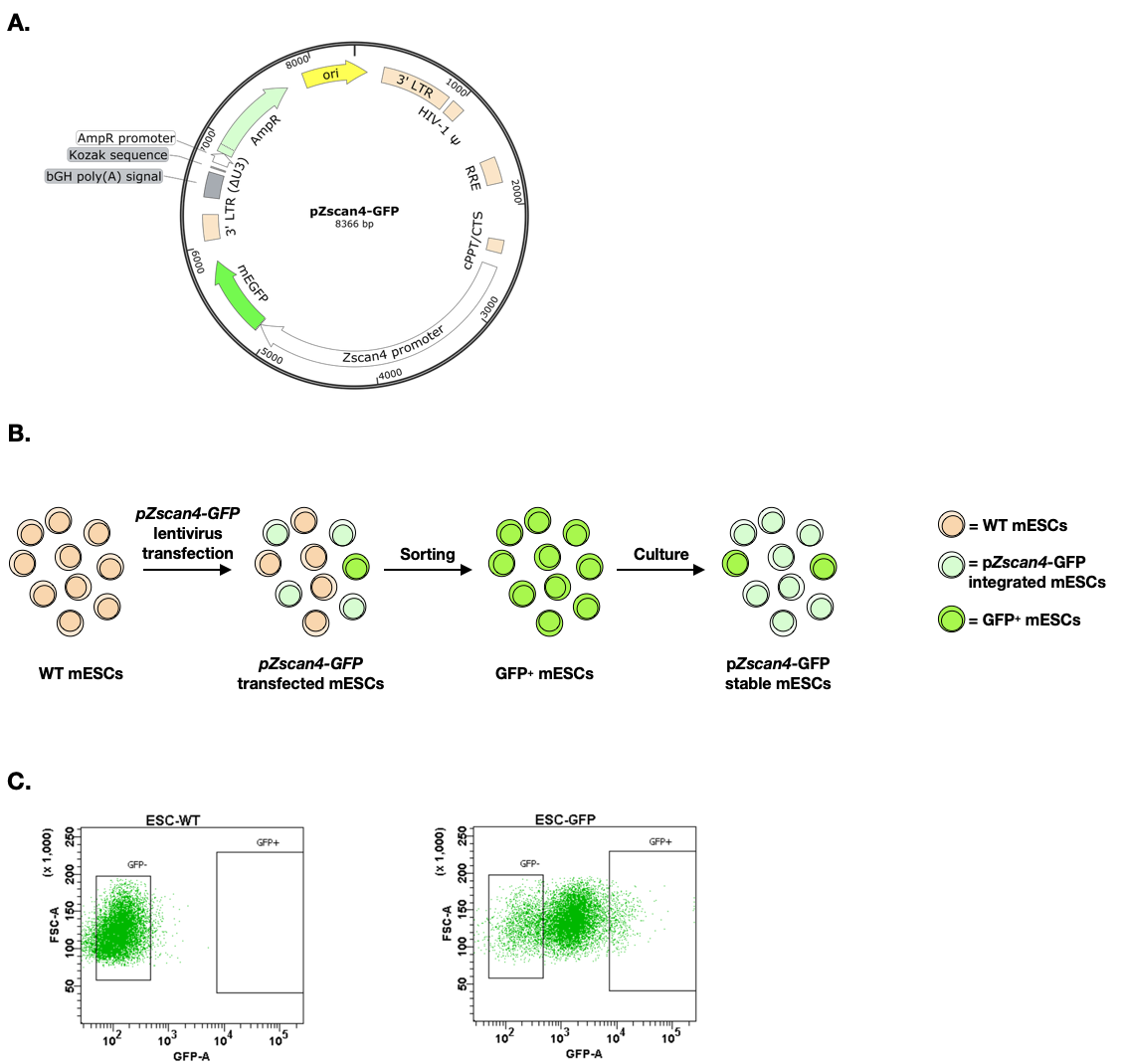


**Figure S2. Plasmid maps of FLAG-ZSCAN4 and HA-PARP1.**

**
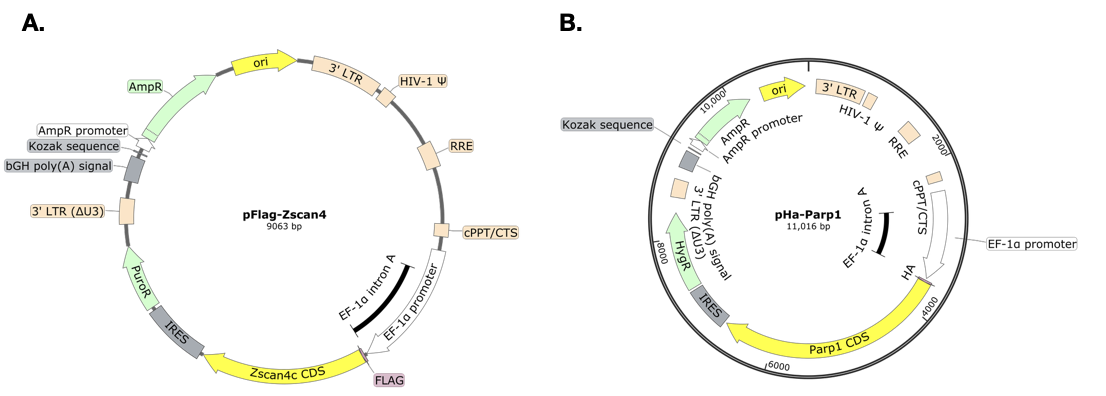
**

**Figure S3. *Parp1* knockout in mESCs.** (A) The map of Parp1 targeting *Cas9* plasmid. The gRNA (CTGGTACCATCCAACTTGCT ) was under U6 promoter. *Cas9* was controlled by chicken β-actin promoter followed by *T2A* and *mCherry*. (B) The map of homologous template for *Parp1* knockout (KO) plasmid. A T2A-GFP-Stop sequence was designed for insertion. (C) Illustration of the *Parp1* knockout strategy. (D) PCR confirmation of the PARP1 knockout. Wildtype (WT) cells had the 208 bp band, whereas cells with the successful knock-in of *T2A-GFP-stop* sequence (which leads to Parp1 KO) had the 993 bp band. WT mESCs and HR template plasmid (PLA) served as control. NC: negative control, water only.

**
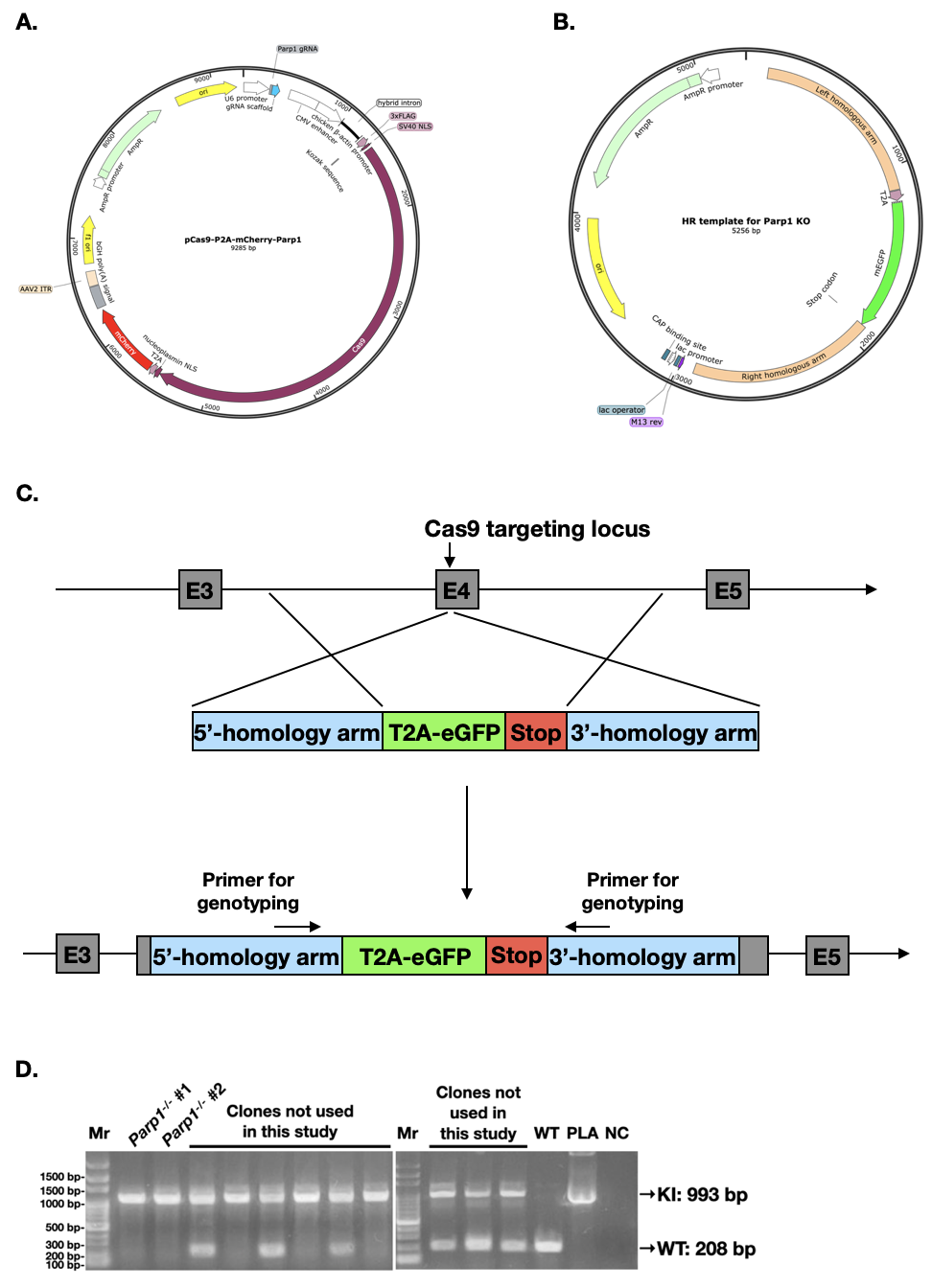
**

**Figure S4. PARP1 binds with ZSCAN4.** Co-IP results of FLAG-ZSCAN4 and HA-PARP1.

**
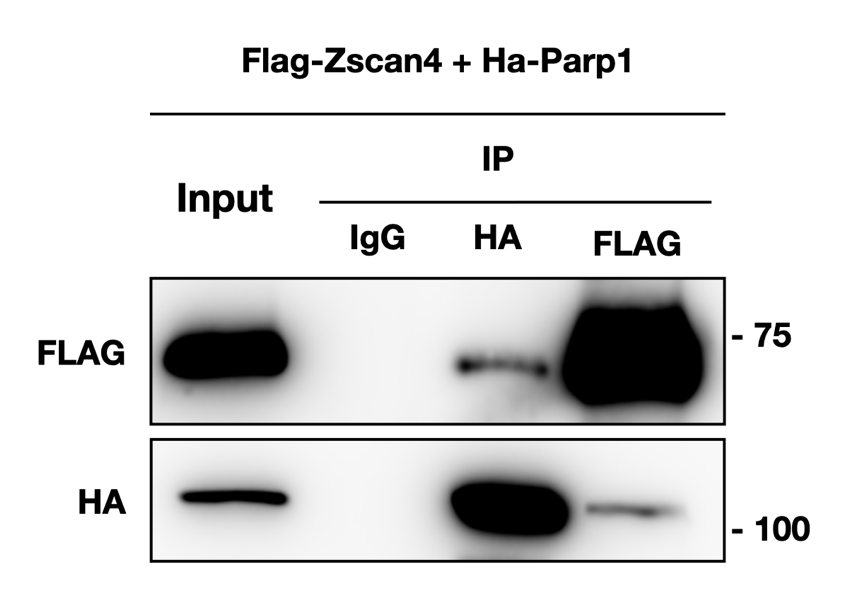
**

**Figure S5. Illustration of the 𝜸H2AX foci counting assay.** The 𝜸H2AX foci (green dots) were counted for each cell. In Example 1, the cell has 7 foci, which is smaller than 10. In Example 2, the cell has 7 foci, which is also smaller than 10. In Example 3, the cell has 23 foci, which is greater than 10. Orange squares indicate the foci counted for the data. Scale bar: 10 µm.


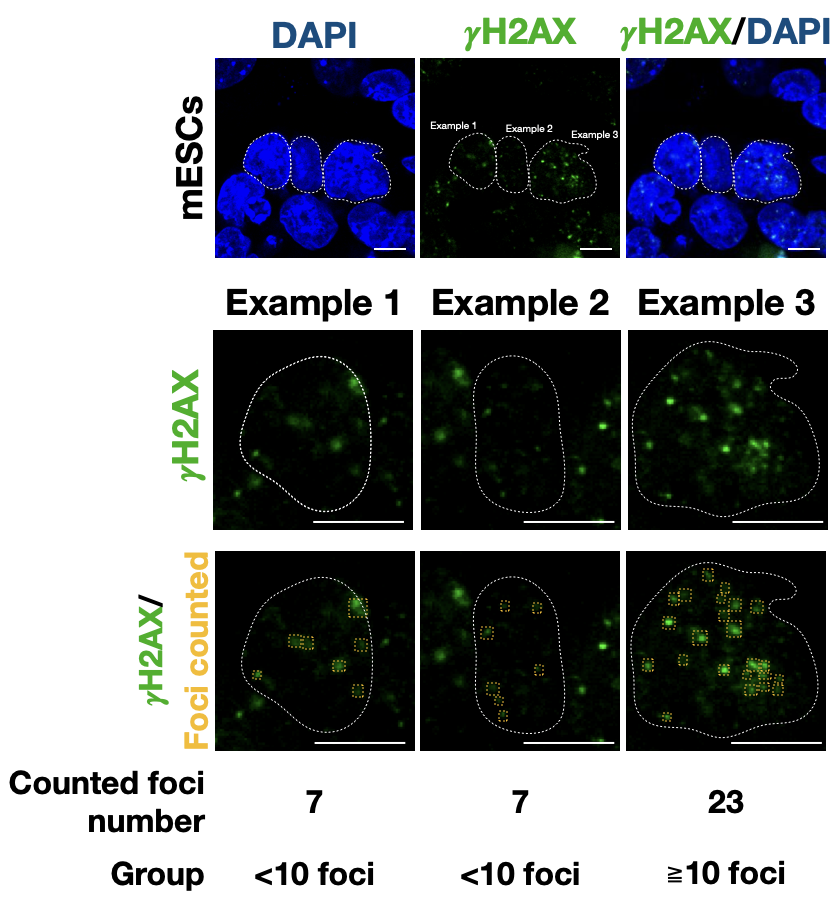


**Table S1. Summary of Co-IP results.**

| Figure 4C |  | FLAG-SCAN | FLAG-LS | FLAG-ZF |  |  |
| --- | --- | --- | --- | --- | --- | --- |
|  | HA-PARP1 | **+** | - | **+** |  |  |
| Figure 4D |  | HA-DB | HA-AM | HA-CAT |  |  |
|  | FLAG-ZSCAN4 | **+** | **+** | - |  |  |
| Figure 4G |  | HA-DB | HA-AM |  |  |  |
|  | FLAG-SCAN | **+** | **+** |  |  |  |
| Figure 4H |  | HA-DB | HA-AM |  |  |  |
|  | FLAG-ZF | - | **+** |  |  |  |
| Figure 5C |  | FLAG-SCAN-△⍺1 | FLAG-SCAN-△⍺2 | FLAG-SCAN-△⍺3 | FLAG-SCAN-△⍺4 | FLAG-SCAN-△⍺5 |
|  | HA-PARP1 | **+** | - | **+** | **+** | **+** |
| Figure 5D |  | FLAG-ZF-△ZF1 | FLAG-ZF-△ZF2 | FLAG-ZF-△ZF3 | FLAG-ZF-△ZF4 |  |
|  | HA-PARP1 | - | - | **+** | - |  |
| Figure 5F |  | FLAG-ZSCAN4  -△⍺2ZF2 | FLAG-ZSCAN4  -△⍺2ZF4 |  |  |  |
|  | HA-PARP1 | **+** | - |  |  |  |

**Table S2. Summary of counted cell numbers in the experiments.**

| Figure 1D | FLAG- | FLAG+ |  |  |  |  |  |  |
| --- | --- | --- | --- | --- | --- | --- | --- | --- |
|  | 274 | 213 |  |  |  |  |  |  |
| Figure 1E | 0% H_2_O_2_ | | 10^-2^% H_2_O_2_ | |  |  |  |  |
|  | FLAG- | FLAG+ | FLAG- | FLAG+ |  |  |  |  |
|  | 405 | 183 | 846 | 129 |  |  |  |  |
| Figure 2B | No 3-AB | | 3-AB | |  |  |  |  |
|  | FLAG- | FLAG+ | FLAG- | FLAG+ |  |  |  |  |
|  | 702 | 190 | 476 | 164 |  |  |  |  |
| Figure 2D | *Parp1^+/+^* | | | | *Parp1^-/-^* | | | |
|  | #1 | | #2 | | #1 | | #2 | |
|  | FLAG- | FLAG+ | FLAG- | FLAG+ | FLAG- | FLAG+ | FLAG- | FLAG+ |
|  | 496 | 246 | 818 | 291 | 665 | 244 | 661 | 236 |
| Figure 6B | FLAG- | | FLAG+ | |  |  |  |  |
|  | Wildtype | △⍺2ZF4 | Wildtype | △⍺2ZF4 |  |  |  |  |
|  | 393 | 424 | 176 | 159 |  |  |  |  |

**Table S3. List of antibodies**

| Antibody | Cat. No. | Vendor | Application (dilution) |
| --- | --- | --- | --- |
| ZSCAN4 | ab4340 | Millipore | IF: 1:500; WB:1:1000 |
| FLAG | F7424 | Sigma | IF: 1:500; WB:1:2000;  IP: 1 µg. |
| FLAG | 66008-4-Ig | Proteintech | IP: 1 µg. |
| 𝜸H2AX | Ab2893 | Abcam | IF: 1:500; WB:1:1000 |
| ACTIN | 23660-1-AP | Proteintech | WB:1:10000 |
| PARP1 | 9542 | Cell signaling | WB:1:1000 |
| HA | Sc-7392 | Santa Cruz | IF: 1:500; WB:1:1000;  IP: 2 µg. |
| Alexa anti-mouse 488 | A11001 | Thermo | IF: 1:500 |
| Alexa anti-rabbit 488 | A11034 | Thermo | IF: 1:500 |
| Alexa anti-mouse 594 | A11032 | Thermo | IF: 1:500 |
| Alexa anti-rabbit 647 | A27040 | Thermo | IF: 1:500 |
| Goat anti-Mouse IgG (H+L) Secondary Antibody, HRP | 31340 | Thermo | WB: 1:6000 |
| Goat anti-Rabbit IgG (H+L) Secondary Antibody, HRP | 31460 | Thermo | WB: 1:6000 |
